# The Mechanisms of *Bacillus subtilis* as a Plant-Beneficial Rhizobacterium in Plant–Microbe Interactions

**DOI:** 10.3390/microorganisms13122823

**Published:** 2025-12-11

**Authors:** Mark Owusu Adjei, Ruohan Yu, Xianming Cao, Ben Fan

**Affiliations:** Co-Innovation Center for Sustainable Forestry in Southern China, College of Forestry and Grassland, Nanjing Forestry University, Nanjing 210037, China; markowusu7@njfu.edu.cn (M.O.A.); yrhnana@163.com (R.Y.); caoxianming2018@gmail.com (X.C.)

**Keywords:** biofilms, biological control, soil rhizobacteria, root colonization

## Abstract

The rhizosphere is a dynamic microenvironment where plants interact with diverse native microbial communities that significantly influence growth, health, and resilience. Among plant-growth-promoting rhizobacteria, *Bacillus subtilis* stands out as a multifunctional species with exceptional ability to colonize plant roots, form robust biofilm, and confer protection against diseases. Its resilience as a spore-former, genetic ability to produce active compounds such as antibiotics, and phytohormones make it a valuable species for agriculture and forest sustainability. This review reveals the molecular and physiological mechanisms underlying *B. subtilis* interactions with plants, focusing on biofilm formation, root colonization, biocontrol and disease suppression, and promotion of plant growth. We further examine its role in root colonization, which triggers extensive reprogramming of plant gene expression, thereby integrating growth promotion with enhanced immune competence through a network that regulates plant-beneficial traits. Its genomic regulation supports colonization, stress tolerance, and immune support, while synergistic interactions with other microbes highlight its adaptability. As a versatile bio-fertilizer and biocontrol agent, further study of its strain-specific traits and rhizosphere interactions is key to maximizing its role in sustainable agriculture and forest control under environmental changes.

## 1. Introduction

The rhizosphere is a biologically active zone surrounding plant roots, where plants, soil, and diverse microbial communities interact dynamically [1]. Within this microenvironment, plants release root exudates that feed and shape microbial assemblages, while microorganisms influence plant development, nutrient acquisition, and stress tolerance. Among these beneficial microbes, plant-growth-promoting rhizobacteria (PGPR) represent a key functional group known to have the ability to enhance productivity and mitigate the impact of diseases through direct and indirect mechanisms [2,3]. The direct mechanism includes the synthesis of phytohormones such as indole-3-acetic acid (IAA), gibberellin, ethylene, cytokinins, and abscisic acid (ABA), which collectively influence root elongation, lateral root formation, shoot development, and stress resilience [4]. Moreover, PGPR increase the availability of essential nutrients by solubilizing inorganic phosphate, producing siderophores that chelate iron, or releasing enzymes that mobilize mineral bound nutrients [5]. Indirectly, plant benefits arise from different ways, including the ability of PGPR to suppress pathogens through the competition for nutrients, producing antimicrobial metabolites, the secretion of lytic enzymes, and activation of plant defense pathways [6,7]. These activities enable rhizobacteria to function as effective bio-stimulants and biological control agents, contributing significantly to sustainable agriculture and forest ecology [8,9].

*B. subtilis* is a typical rhizobacteria species that plays a critical role in promoting plant health, stimulating growth, and enhancing resistance to disease [10]. Due to its generally regarded safety and endospore-forming ability, *B. subtilis* has become one of the most extensively studied and widely commercialized plant-associated bacteria in agricultural application [11,12]. Most *Bacillus* strains produce diverse antibiotics and hydrolytic enzymes as effective biocontrol agents against pathogenic fungi, thereby contributing to disease suppression [13,14]. Their other beneficial effects involve biofilm formation, which, as a physical barrier, separates pathogens from plant roots, the systemic induction of hosts resistant to phytopathogens, and other activities outlined above for PGPR [15,16]. Over the years, *B. subtilis* has expanded its role as a model organism for the study of the fundamental biology of PGPR and Gram-positive bacteria, and a popular practicable species used as a bio-fertilizer, bio-stimulant, or biocontrol agent for sustainable agriculture [17].

**Figure 1:**
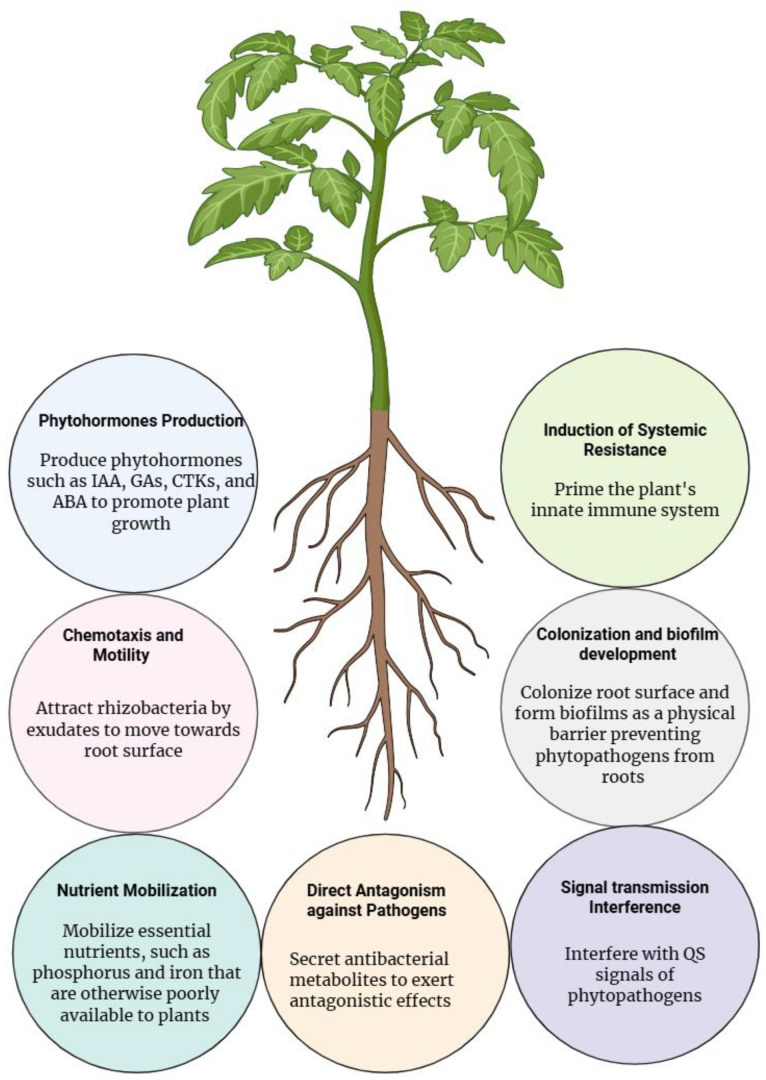
A comprehensive schematic illustration of the multifaceted plant-growth-promoting activities of *B. subtilis*, including (I) phytohormone biosynthesis (e.g., IAA, GAs, CTKs, ABA), modulating plant growth and development processes; (II) direct chemotaxis along root exudate gradients, enabling targeted rhizosphere colonization; (III) nutrient solubilization and mobilization (notably phosphorus and iron), increasing bioavailability of essential minerals; (IV) induction of systemic resistance (ISR), priming the plant’s innate immune defenses; (V) robust root colonization and structural biofilm development, forming a competitive physical barrier against pathogen ingress; (VI) direct antagonism via the production of specialized antimicrobial secondary metabolites; and (VII) quorum sensing (QS) interference, disrupting pathogenic communication and virulence.

Numerous studies have revealed that *B. subtilis* interacts with plants through complex regulatory networks that influence biofilm development, chemotaxis, metabolite production, and signal exchange with the host. These interactions modify plant transcriptional programs associated with growth, nutrient uptake, and immunity, thereby integrating multiple beneficial traits into coordinated plant–microbe relationships [18,19]. In this review, we summarize the molecular, physiological, and ecological mechanisms that underlie *B. subtilis*–plant interactions. We discuss their roles in root colonization, biofilm formation, nutrient acquisition, hormone modulation, and biocontrol, as well as the regulatory network governing these traits (Figure 1). By integrating current knowledge, we highlight potential *B. subtilis* as a versatile PGPR and identify research gaps that must be addressed to optimize its application in sustainable plant and forest management.

## 2. Biofilm Formation and Root Colonization

### 2.1. Cell Heterogeneity and Biofilm Development

**Figure 2:**
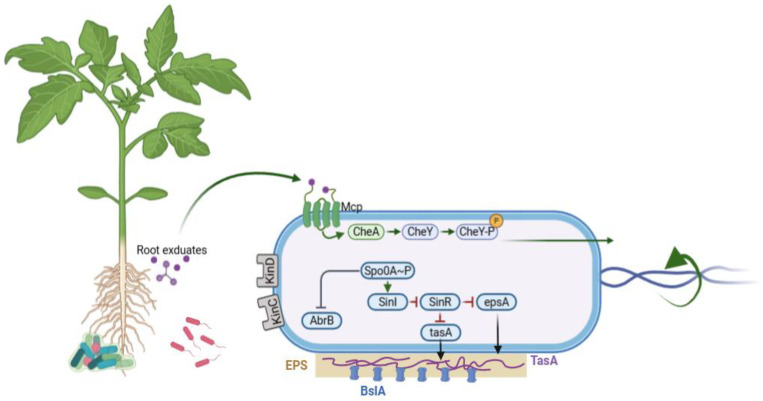
Model of the regulatory network governing chemotaxis, motility, and biofilm formation in *B. subtilis* response to root exudates. Roots exudates (environmental) are sensed via the chemotaxis system, leading to phosphorylation of the response regulator CheY (ChenY~P), which promotes motility inhibition. The phosphorylated master regulator Spo0A~P activates biofilm formation by relieving repression of the matrix operons. The SinI–SinR antirepressor system further fine-tunes the expression of the exopolysaccharide (EPS) gene cluster (*epsA-O*) and the amyloid fiber gene *tasA*. Downstream, the hydrophobin BslA aids in structuring the biofilm surface.

The first step in plant–microbe interactions is the successful colonization of rhizosphere and root surface [20]. *B. subtilis* forms structured biofilms mediated by extracellular matrix components such as exopolysaccharides (EPSs) and extracellular matrix (ECM), TasA amyloid fibers, and BslA hydrophobin-like proteins. Among these, EPS and TasA are particularly essential for effective root colonization [10]. Studies show that *B. subtilis* mutants lacking EPS (Δeps) or TasA (ΔtasA) have significantly reduced colonization ability. However, when these mutants are co-inoculated, biofilm formation and colonization are restored, comparable to the wild type, suggesting resource sharing and task division within the bacterial community (Figure 2) [17].

Mature *B. subtilis* biofilms display remarkable cellular diversity, consisting of not only matrix-producing cells but also competent, motile, sporulating, cannibalistic, and mining cells [21]. This cellular heterogeneity supports a cooperative structure that optimizes the resource use and benefits of the overall microbial community. The differentiation and behavior of these cell types are tightly regulated by three master regulators: DegU, ComA, and Spo0A [18,19]. DegU controls exoprotease secretion, ComA regulates competence and surfactin production, and Spo0A controls matrix production and sporulation. Spo0A plays a central role in the transition from motility to sessile biofilm formation, largely through its effect on the SlrR/Sinl/SinR regulatory pathway [22]. At intermediate phosphorylation levels, Spo0A activates SinI, which inhibits SinR, allowing for the expression of *slrR* and thereby promoting matrix gene expression [23]. A positive feedback loop between SinR and SlrR maintains this matrix active state. High Spo0A~P levels, however, inhibit SinI and promote sporulation, while low levels allow for the repression of matrix genes by *abrB* [19].

Five distinct kinases (KinA to KinE) initiate the phosphorylation cascade of Spo0A in *B. subtilis.* They do this by responding to various environmental and host signals [24]. Among these, KinC and KinD have been shown to directly influence root colonization by initiating biofilm production. A mutant strain lacking the *kinD* gene was unable to form biofilms on *Solanum lycopersicum* (*S. lycopersicum*) roots, and it was determined that L-malic acid, a root exudate, acts as a signal for biofilm formation, likely serving as a carbon source to support biofilm development [25]. This process strongly depends on the sensor kinase KinD and, to a lesser extent, KinC, highlighting their central role in mediating plant microbe signaling [26]. The role of KinC and KinD in biofilm formation was further confirmed through studies on plant polysaccharides, including arabinogalactan and pectin [27]. While KinC and KinD were found to be responsible for detecting arabinogalactan and pectin, all mutants, including KinC and KinD gene knockout, still formed biofilms in response to xylem, suggesting the existence of an unknown mechanism for biofilm induction by certain plant stimulants [21]. *B. subtilis* strains with null mutations in Spo0A regulatory genes exhibit either enhanced colonization (Δ*abrB* and Δ*sinR)*, due to robust biofilm formation, or impaired colonization (Δ*sinI*, Δ*eps*, and Δ*tasA*), due to defective matrix production [28,29,30]. Research on *S. lycopersicum* roots confirmed that these biofilm regulatory genes are essential not only for colonization but also effective biocontrol against pathogens such as *Ralstonia solanacearum* (*R. solanacearum*) [31]. The ability of *B. subtilis* to colonize plant roots and form biofilms is governed by complex regulatory networks that integrate environmental evidence and coordinate cellular differentiation [32]. Once inside the cell, Phr peptides inhibit Rap phosphatases, which, in turn, activate the master regulators (DegU, ComA, and Spo0A), leading to changes in genes and the stabilization of the cooperative behavior essential for biofilm formation and root colonization (Figure 2) [33].

### 2.2. Chemotaxis and Motility

During the early stage of root colonization, *B. subtilis* greatly rely on chemotaxis to establish themselves effectively on plant roots. During this process, chemotaxis allows bacteria to detect chemical gradients and migrate towards a beneficial environment or away from harmful substances [6,7]. This response is triggered when attraction molecules bind to bacterial chemoreceptors, activating the CheA kinase, which then phosphorylates the response regulator CheY [34]. Phosphorylated CheY controls the rotation of the flagella motor, enabling the transition between swimming (counterclockwise) and tumbling (clockwise rotation). In *B. subtilis*, chemotaxis is crucial for successful root colonization [35]; however, its movement is not directed but rather a random, biased stroll, which enhances the bacterium’s ability to reach favorable areas [36].

Mutants lacking chemotaxis genes (Δ*cheA*, Δ*hag*, and Δ*motA)* are unable to establish colonies on plant roots, emphasizing the crucial role played by chemotaxis in root attachment [28,29,30]. In response to root exudates, several chemoreceptors, including McpA, McpB, and McpC, have been identified (Figure 2), with McpA showing affinity for compounds in the exudates that may act as repellents [37]. Specific root exudates, such as L-malic acid, can enhance *B. subtilis* colonization in *Arabidopsis thaliana* (*A. thaliana*) by triggering the release of compounds in response to pathogen infection [24]. Additionally, *B. subtilis* displays stronger chemotactic attraction to host plant root exudates (e.g., banana) than to those of non-hosts (e.g., cucumber), indicating host-specific chemotactic responses [38,39]. While chemotaxis is essential in liquid environment, its role in soil colonization is debated. The swimming movement involves a group of cells that travel collectively across a solid surface, and they play a larger role in the natural soil system. Unlike chemotaxis, swarming does not rely on directed movement but requires surfactin synthesis [40]. Comparisons between chemotaxis mutants (Δ*cheV*) and swarming-deficient mutants (Δ*srfAC*, Δ*swrA*, and Δ*minJ*) revealed that, although chemotaxis contributes to root colonization, swarming appears to be more critical, as mutants unable to swarm showed significantly reduced colonization [41]. Together, chemotaxis and swarming play important roles in the colonization of plant roots by *B. subtilis*, with chemotaxis primarily facilitating movement towards beneficial root exudate and swarming, promoting effective colonization in soil.

## 3. Biocontrol and Disease Suppression

### 3.1. Signal Transduction Interference

**Figure 3:**
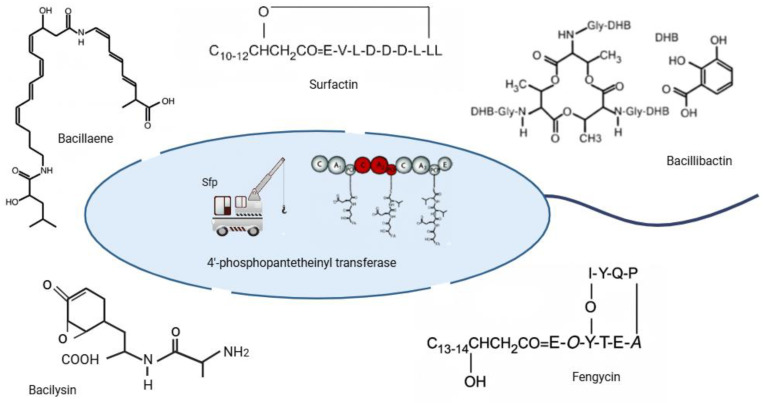
Antibiotics with biocontrol activity produced by *B. subtilis* strain NCBI 3610. Surfactin, a cyclic heptapeptide linked to a C_10_–C_12_ β-hydroxy fatty acid, acts as a powerful bio-surfactant and elicitor of systemic resistance in plants. The fengycin, a decapeptide with a C_13_–C_14_ β-hydroxy fatty acid tail, exhibits potent antifungal activity by disrupting membrane integrity. Bacilysin, a dipeptide antibiotic composed of L-alanine and L-anticapsin, irreversibly inhibits glucosamine synthase and shows broad-spectrum antibacterial and antifungal properties. With the exception of bacilysin, the biosynthesis of all four other antibiotics depends on 4′-phosphopantetheinyl transferase (Sfp).

Many phytopathogens rely on quorum sensing (QS) signals, particularly N-acyl homoserine lactones (AHLs), to activate virulence related genes. This suggests that interference with these QS signals offers an effective strategy to mitigate disease severity [42]. *B. subtilis* has emerged as a quorum-quenching (QQ) bacterium due to its ability to produce N-acyl homoserine lactonases that inactivate AHLs, thereby disrupting pathogen communication (Figure 3) [32]. The most widely characterized gene is a*iiA* (it encodes the aiiA enzyme, which is a metallo-β-lactonase), first identified in *B. subtilis* strain 168, which encodes a metalloprotease-like lactonase capable of hydrolyzing the homoserine lactone ring of diverse AHL molecules. Functional assays have demonstrated that *B. subtilis* harboring a*iiA* effectively degraded both short- and long-chain AHLs, blocking QS-dependent regulation of pathogenicity genes. Similarly, the *ytnP* gene encodes another lactonase in *B. subtilis* UD1022, and its expression has been linked to the suppression of AHL-regulated traits in plant pathogens [43]. Practical applications of these QQ activities have been reported in plant protection studies. For example, *B. subtilis* strain BS-1, carrying an N-acyl homoserine lactonase enzyme, significantly reduced potato soft rot caused by *Erwinia carotovora*, a pathogen whose virulence is tightly controlled by AHL-mediated QS. Furthermore, the expression of a*iiA* in recombinant *B. subtilis* and related strains consistently decreased AHL levels in vitro and reduced disease symptoms, reinforcing the central role played by this enzyme in QS interference. The ability of *B. subtilis* lactonases to disrupt pathogen signaling highlights their potential use as biocontrol agents (Figure 3) [44].

### 3.2. Direct Antagonism Against Pathogens

*B. subtilis* exerts direct antagonism against diverse plant pathogens through the secretion of antimicrobial metabolites, such as the lipopeptides surfactin, iturin, and fengycin. These bioactive compounds disrupt pathogen membranes, inhibit spore germination, and suppress hyphal growth [20]. Surfactin, a cyclic lipopeptide produced by *B. subtilis*, functions as a key secondary metabolite with roles in cell signaling [45] and surface tension reduction [40]. The death rates of *A. thaliana* after *Pseudomonas syringae (P. syringae*) infection decreased when inoculated with surfactin. However, inoculation on a mutant strain without surfactin had no effect [5]. This suggests that surfactin may effectively stop *P. syringae* growth. Nevertheless, surfactin-deficient mutants have exhibited significant phenotypic alterations that might potentially reduce their ability to control biological organisms. Moreover, due to the strong interconnection between the production of surfactin and the synthesis of other secondary metabolites that possess antibacterial capabilities, it is possible that the mutant strain may also be deficient in other antimicrobials [46]. Under lab conditions, *B. subtilis* 9407 strain producing surfactin had antibacterial effects against *Acidovorax citrulli* (*A. citrulli*) [47]. In greenhouse assays, a *B. subtilis* strain effectively controlled *A. citrulli* on melon seedlings [48].

More importantly, surfactin and bacillomycin together in a synergistic manner can suppress some plant infections because of interconnected biosynthesis pathways [49]. It has been demonstrated that *B. subtilis* mutants with impaired surfactin synthesis did not produce bacillomycin [50], but *B. subtilis* mutants that lack the ability to produce bacillomycin have reduced efficacy in controlling *Rhizoctonia solani* compared to the wild type [50,51]. *B. subtilis* produces exoenzymes such as proteases and chitinases to break down the fungal infection cell wall. The exoenzyme chitinase generated by *B. subtilis* is the primary antifungal compound [52]. In *S. lycopersicum* seedlings, a chitinase-producing *B. subtilis* strain decreased the number of damaged plants from 20% and 35%, as observed in greenhouse and field experiments, respectively [53]. Moreover, *B. subtilis* can prevent the spore germination and hyphal development of the plant pathogen *Botrytis cinerea* on agar plates without the need for direct contact. The capacity of *B. subtilis* to demonstrate biocontrol efficacy relies on three factors, mainly host susceptibility [54], pathogen virulence [44], and environmental conditions [8,9] (Figure 3).

### 3.3. Induction of Systemic Resistance

*B. subtilis* not only engages in quorum-quenching but also protects plants by ISR, a defense mechanism in which non-pathogenic rhizobacteria prime the plant’s innate immune system for enhanced protection against subsequent pathogen attacks (Table 1) [55]. Several metabolites and proteins secreted by *B. subtilis* act as ISR elicitors. Among them, lipopeptides such as surfactin, iturin, and fengycin trigger early defense responses including reactive oxygen species production, cell wall fortification, and activating defense-related genes [56]. Volatile organic compounds (VOCs), notably *2*,*3-butanediol* and acetoin, modulate JA/ET signaling to further stimulate ISR [4]. Additionally, microbe-associated molecular patterns (MAMPs) such as flagellin (encoded by *hag*) and bacterial lipoproteins are recognized by plant pattern recognition receptors (PRRs), leading to ISR priming [56]. Strain-specific studies highlight the ISR potential of *B. subtilis* such as GB03 that produces acetoin and *2*,*3-butanediol*, inducing ISR in *A. thaliana* and *S. lycopersicum* [55]. Strain FB17 triggers JA/ET-dependent ISR in *A. thaliana*, enhancing resistance to *P. syringae* and QST713, through surfactin production, promoting ISR across various plants [57]. Upon ISR induction, plants exhibit the upregulation of key defense-related genes where *PDF1* gene serves as a marker for JA/ET-mediated ISR, while Pathogenesis-Related Protein 3 and 4 (*PR3* and *PR4*) genes encode chitinases that degrade fungal cell walls [58,59]. *LOX2* is crucial for JA biosynthesis, and *ERF1* links the JA [60] and ET pathways [61]. This gene activation equips plants with the help of *B. subtilis* to resist a broad spectrum of pathogens, *Fusarium*, *Botrytis* [40], and *R. solanacearum* [5] (Table 1). When *B. subtilis* colonizes the root system, it elicits plant defense responses throughout the entire plant, extending protection even to tissues where the bacteria are absent (Figure 2 and Figure 3). This form of resistance induces a heightened state of alertness, enabling the plant to mount faster and stronger defenses against diverse pathogens.

**Table 1:** *B. subtilis* biocontrol and disease suppression in plants.

Species	Tissues	Infection	Strains	Mechanisms/Mode of Action	Ref.
*Arabidopsis thaliana*	Stomata	*Pseudomonas syringae*	*B. subtilis FB17*	Surfactin-mediated ISR, stomatal closure, and biofilm formation	[5]
*Arabidopsis thaliana*	Roots	*Pseudomonas syringae*	*B. subtilis 6051*	Surfactin secretion and root biofilm formation	[62]
*Solanum lycopersicum*	Roots	*Pseudomonas syringae* pv *tomato* DC3000	*B. subtilis IAB/BS03*	Lipopeptide production (surfactin/iturin) and ISR pathway activation	[63]
*Solanum lycopersicum*	Leaf	*Pseudomonas syringae* pv. and *Alternaria solani*	*B. subtilis*	Systemic resistance interconnected via JA/ET pathways	[55]
*Solanum tuberosum*	Whole plants	*Potato virus Y*	*B. subtilis EMCCN 1211*	Antiviral defense activation and reduction in PVY accumulation	[64]
*Phaseolus vulgaris*	Root	*Pythium aphanidermatum*	*B. subtilis HE18*	ISR activation, root growth improvement, and degradation of pathogen structures	[65]
*Passiflora edulis Sims*	Stem and plant	Leaf Blight	*B. subtilis GUCC4*	Antifungal metabolite production and colonization of stem tissues	[66]
*Solanum lycopersicum*	Roots and leaves	*Alternaria solani*	*B. subtilis J3*	Regulation of resistance genes and antifungal secondary metabolites	[67]
*Cucumis melo*	Roots and leaves	*Acidovorax citrulli*	*B. subtilis 9407*	Surfactin-mediated inhibition of Acidovorax biofilms; suppression of swarming motility	[47]
*Solanum lycopersicum*	Sprouting and seedling	*Rhizoctonia* rot	*B. subtilis SL-13*	Chitinase secretion; inhibition of *Rhizoctonia*; growth promotion	[68]
*Oryza sativa*	Rice sheath	*Rhizoctonia solani*	*B. subtilis 916*	Production of bacillomycin/surfactin; suppression of *R. solani*; reduced biofilm required for pathogenic attack	[50]
*Arabidopsis thaliana*	Seedling leaves	*Erwinia carotovora*	*B. subtilis*	Quorum-quenching, AHL degradation, VOC-mediated ISR	[69]

## 4. Promotion of Plant Growth

### 4.1. Phytohormone Production

*B. subtilis* promotes plant growth by producing phytohormones such as IAA, GAs, cytokinins, and ABA, thereby modulating root architecture, shoot development, and stress resilience (Table 2) [6,7]. Auxin production by *B. subtilis* has been extensively characterized, with strains such as GB03 and FB17 promoting root elongation, lateral root formation, and enhanced nutrient uptake in *A. thaliana* and *S. lycopersicum* [70]. These phenotypic changes correlate with the transcriptional activation of auxin-responsive genes, including Auxin Response Factor (ARF) and IAA family members, underscoring a direct link between bacterial auxin biosynthesis and plant developmental programming [13,14]. Similarly, GA-producing strains, including *B. subtilis* BSF01 and *B. subtilis* QST713, enhance stem elongation, leaf expansion, and seed germination through the upregulation of *GA20ox* and *GA3ox*, key enzymes in endogenous GA biosynthesis (Table 2). Cytokinins producing *B. subtilis* strains, exemplified by *B. subtilis BSn5*, modulate cell division and delay senescence, thereby improving photosynthetic efficiency and biomass accumulation. Concurrently, ABA synthesis by *B. subtilis* strains enhances plant tolerance to abiotic stresses, mediated through stomatal regulation and the induction of stress-responsive genes (Table 2) [71]. Moreover, some strains ameliorate ethylene-induced growth inhibition by producing ACC deaminase, which break down the ethylene precursor ACC [72]. This reduction in ethylene levels promotes root elongation, enhances nutrient uptake [73], and improves tolerance to abiotic stresses, including salinity [56] and drought [74]. Notably, the combination effect of these phytohormones often results in the synergistic promotion of plant growth and resilience, highlighting the sophisticated capacity of *B. subtilis* to integrate hormonal signaling pathways with environmental cues [28,29,30]. These insights position *B. subtilis* not merely as a passive rhizosphere inhabitant but as a dynamic bioengineering agent, capable of modulating plant hormonal networks at both physiological and transcriptional levels. Harnessing such microbial-mediated phytohormone production offers a promising avenue for plant improvement, bridging molecular understanding with translational potential (Figure 3).

### 4.2. Nutrient Mobilization

*B. subtilis* enhances plant growth and productivity by mobilizing essential nutrients, such as phosphorus and iron that are otherwise poorly available to plants (Table 2). Through the secretion of organic acids, siderophores, and phosphatases, *B. subtilis* mobilizes insoluble phosphates and ferric complexes, converting them into forms readily accessible to plant roots [75]. In most cases, mobilization of these essential nutrients is dependent on two factors consisting of the phosphorus essential factor and siderophore production factor [74]. Phosphorus is essential for supporting root growth, energy transfer (ATP), and nucleic acid synthesis. However, most soil phosphorus is present in mineral-bound, insoluble forms that roots cannot access. When colonizing plant roots, *B. subtilis* secretes organic acids, *Glucose dehydrogenase*, which release soluble phosphate into the rhizosphere [75]. Strain-specific studies highlight the effectiveness of nutrient mobilization (Table 2).

For instance, *B. subtilis* GB03 and FB17 enhance phosphorus uptake in *A. thaliana* and *Nicotiana tabacum* (*N. tabacum*) [74], correlating with increased root surface area and biomass. These changes are associated with the upregulation of plant phosphate transporter genes, such as *Phosphate Transporter 1* (*PHT1*) and *Phosphate 1* (*PHO1*), which facilitate efficient phosphate acquisition. Similarly, siderophore-producing strains like *B. subtilis GB03* [76] and *B26* [75] improve iron availability and induce the expression of iron-regulated transporter genes *Iron-Regulated Transporter 1* (*IRT1*) and *Ferric Reductase Oxidase 2* (*FRO2*), promoting chlorophyll biosynthesis, photosynthetic efficiency, and overall plant vigor (Figure 1). This process benefits plants by improving iron uptake for growth and metabolism. At the same time, by tightly competing for iron, *B. subtilis* restricts pathogen access to this vital nutrient, indirectly protecting plants from infections [77]. This is primarily achieved through the secretion of organic acids and enzymatic activity, which convert these nutrients into forms that can be readily absorbed by plant roots [72].

**Table 2:** *B. Subtilis* in modulating plant hormone responses to promote growth.

Species	Tissues	Strains	Effect on Plants	Hormones Implicated	Ref.
*A. thaliana*	Leaves	*B. subtilis*	Improved stomatal responsiveness	Ethylene modulation via VOCs (2,3-butanediol)	[78]
*A. thaliana*	Leaves and roots	*B. Subtilis GB03*	Balanced auxin distribution and increased lateral root formation	IAA (auxin)	
*A. thaliana* and *T. aestivum*	Seedlings	*B. subtilis J-15*	Increased shoot biomass and delayed leaf senescence	Cytokinins and ethylene	[79]
*N. tabacum*	Root cells	*B. subtilis OKB105*	Enhanced root elongation and increased cell expansion	Ethylene	[72]
*Lettuce*	Shoots and roots	*B. subtilis*	Higher biomass and delayed senescence	Cytokinins	
*A. thaliana*	Leaf surface area	*B. Subtilis GB03*	Increased leaf area and higher photosynthetic capacity	Cytokinins and ethylene	
*Trigonella foenum-graecum*	Seedlings	*B. subtilis ER-08 (BST)*	Improved tolerance to salt/drought stress; increased biomass	ABA, cytokinins, ethylene	[61]
*A. thaliana*	Seedlings	*B. subtilis GOT9*	Enhanced drought and salt tolerance	ABA, ethylene	[74]
*T. aestivum*	Seedlings	*B. subtilis NA2 strain*	Growth improvement under salinity	ABA, ethylene	[80]
wheat plants	Seedlings	*B. strains NMCN1 and LLCG23*	Improved chlorophyll content; enhanced root architecture under stress	ABA	[81]
*O. sativa* L.	Seedlings	*B. subtilis*	Enhanced drought and salt tolerance; ABA-dependent regulation	ABA	[82]
*H. vulgare* L.	Roots	*B. subtilis IB22*	ABA-dependent growth improvement under salt stress	ABA	[56]
*Brachypodium distachyon*	Seedlings	*B. subtilis B26*	Increased biomass; enhance	ABA	[75]
*Arabidopsis thaliana*	Seedlings	*B. Subtilis GB03*	Improved osmotic stress tolerance via VOC signaling	IAA, ABA interaction	[4]
*Saccharum* spp.	Roots and stalk	*B. subtilis*	Improved nutrient uptake; enhanced drought resilience	ABA, cytokinins	[73]
*Phleum pratense*	Seedlings, roots and shoots	*B. subtilis B26*	Increased root/shoot biomass under drought	ABA	[76]
*Brassica napus*	Seedlings	*B. subtilis XF-1*	Improved resistance to *Plasmodiophora brassicae*; healthier roots	IAA, ethylene, JA	[60]

## 5. The Network and Regulation of Plant-Beneficial Traits

### 5.1. Connections Among Plant-Beneficial Traits

**Figure 4:**
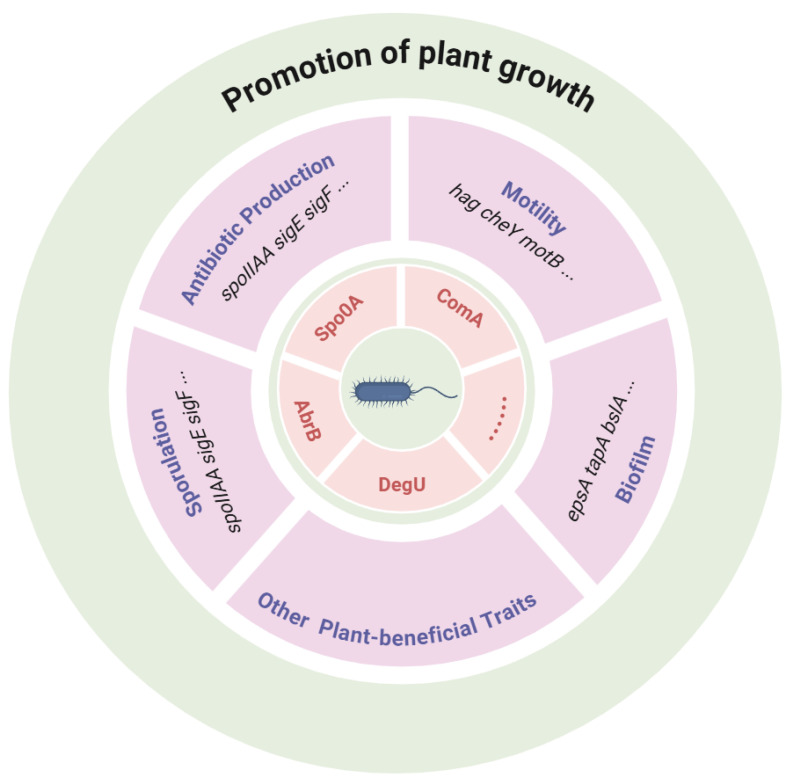
A schematic illustration of major regulators governing plant-beneficial traits of *B*. *subtilis*. The regulators include Spo0A, DegU, ComA, AbrB, and many other global transcriptional regulators. They modulate the expression of genes involved in different mechanisms promoting plant growth, such as chemotaxis, motility, biofilm formation, antibiotic production, etc., which have been discussed above. While the regulations are interconnected and complex, the details have been unraveled in numerous research papers.

Root colonization by *B. subtilis* triggers the extensive reprogramming of plant gene expression, integrating growth promotion with enhanced immune competence [20]. Successful colonization requires the coordinated activation of plant genes involved in cell wall remodeling, hormone signaling, and defense, establishing a dynamic molecular dialog between the host and microbe (Figure 4). The signaling that triggers biofilm development on the root is not one way from the plant to the bacterium but rather a dynamic interaction between both [19]. Besides the plant-synthesized chemicals that induce a chemotactic response and the production of biofilms in bacterial cells, *B. subtilis* also has the ability to modulate gene expression in plants, hence facilitating root colonization [56]. Auxin-producing strains such as *B. subtilis* GB03 and FB17 modulate root architecture by upregulating auxin-responsive genes, including *ARF* and *IAA* family members, promoting lateral root formation and root hair development [6,7]. These morphological changes enhance rhizosphere exploration, nutrient uptake, and microbial colonization, creating a feedback loop that supports both microbial persistence and plant growth [57]. This is accompanied by the transcriptional upregulation of defense-related genes such as *Plant Defensin 1* (*PDF1*), Pathogenesis-Related Protein 3 (*PR3*) and Pathogenesis-Related Protein 4 (*PR4*), *Lipoxygenase* 2 (*LOX2*), and *Ethylene Response Factor 1* (*ERF1*) [80], which collectively strengthen the plant’s ability to resist fungal, bacterial, and viral pathogens (Figure 4, Table 3). Phosphate transporter gene *PHT1* and iron-regulated genes *IRT1* and *FRO2* are upregulated in response to the microbial solubilization of phosphorus and iron, linking nutrient acquisition with hormone- and ISR-mediated pathways (Figure 3). This coordinated modulation of growth, immunity, and nutrient-responsive genes illustrates a sophisticated regulatory network in which *B. subtilis* acts as both a growth-promoting and defense priming agent [83]. In addition, a protein called *Expansin_EXLX1-like*, which is synthesized and released by *B. subtilis*, appears to play a significant role in plant microbial interactions. The structure of this compound closely resembles that of plant β-expansins [84]. It has the ability to attach to plant cell walls and stimulate their growth. Furthermore, the study demonstrates that mutants with a deficiency in *Expansin_EXLX1-like* synthesis exhibited a notable reduction in root colonization when compared to the wild type [60]. Collectively, these findings underscore the role of *B. subtilis* in orchestrating molecular and genetic regulatory networks in plants, integrating root development, nutrient acquisition, and systemic immunity (Table 3). Such strain-specific, gene-level modulation highlights the potential of harnessing rhizobacteria interactions for plant improvement, bridging fundamental molecular insights with translational agricultural applications.

**Table 3:** Genetic elements of *B. subtilis* underlying its plant-beneficial traits.

Category	Genes/Operons	Function	Plant-Beneficial Role	Ref.
Global Regulators	*Spo0A*, *DegU*, *ComA*, *SinR*, *SinI*	Master transcriptional regulators	Coordinate sporulation, biofilm formation, and secondary metabolite production	[19]
Biofilm Formation and Colonization	*EpsA*, *TapA*, *SipW*, *and TasA*	Extracellular polysaccharides and amyloid fibers	Root colonization, biofilm stability, pathogen exclusion	[17]
	*Tli*, and *Mot*	Flagella and motility proteins	Rhizosphere migration and root attachment	[21]
	*Che*	Chemoreceptors and signaling proteins	Chemotaxis toward plant root exudates	[41]
Secondary Metabolite Clusters	*Srf*	Surfactin synthetase	Biofilm induction, ISR, antimicrobial activity	
	*Itu*, and *Fen*	Iturin, fengycin, plipastatin synthetases	Strong antifungal activity and pathogen suppression	[20]
	*Bac*	Bacilysin biosynthesis	Broad-spectrum antibacterial and antifungal effects	
	*Alb*	Subtilosin A	Antimicrobial peptide	
	*Dhb*	Bacillibactin (siderophore) biosynthesis	Iron acquisition and competition with pathogens	
Plant Growth Promotion	*Gcd*	Glucose dehydrogenase	Organic acid secretion and phosphate Mobilization	[56]
	*YsnE*, and *IpdC*	Auxin (IAA) biosynthesis enzymes	Root growth promotion and architecture modulation	
	*AlsS*, and *AlsD*	Acetoin and 2,3-butanediol biosynthesis	VOC-mediated plant growth stimulation and stress tolerance	[28,29,30]
	*GabT*, and *GabD*	GABA metabolism	Plant–microbe signaling and stress modulation	
Signal Integration and Regulation	*ComX*, *ComP*, and *ComA*	Quorum-sensing system	Controls competence and metabolite biosynthesis	[42]
	*Phr* peptides	Quorum-sensing feedback modulators	Fine-tune population behavior in rhizosphere	
	*DegS*, *DegU*, *PhoP*, *PhoR*, *ResD* and *ResE*	Sensor–regulator systems	Adaptation to nutrient availability, oxygen, phosphate, and plant signals	[28,29,30]
Stress Response and Adaptation	*KatA*, and *SodA*	Catalase and superoxide dismutase	Protection against oxidative stress in rhizosphere	[32]
	*SigB*	General stress sigma factor	Global response to abiotic stress	[6,7]
	*YvcC*	Cell wall modification protein	Adaptation to plant-imposed stresses	

### 5.2. Genes and Their Regulation of Plant-Beneficial Traits

*B. subtilis* functions as a central hub in the rhizosphere, coordinating complex molecular and genetic networks that regulate plant growth, nutrient acquisition, and immunity. Its effects are not limited to direct plant interactions; rather, *B. subtilis* acts synergistically with other beneficial microbes, amplifying physiological and transcriptional responses in the host (Table 3). For example, IAA- and GA-producing strains such as *B. subtilis* GB03 [4] and *B. subtilis* FB17 enhance root architecture by upregulating auxin-responsive genes, promoting lateral root formation and root hair development [19]. When combined with phosphate- and iron-solubilizing microbes, these hormonal effects are reinforced, resulting in the coordinated activation of nutrient transporters *IRT1* and *FRO2*, which improves nutrient uptake and overall plant vigor.

The plant-beneficial potential of *B. subtilis* has a diverse and interconnected genetic toolbox. Global regulators such as Spo0A, DegU, ComA, and SinR act as master switches, coordinating sporulation, biofilm development, and metabolite production (Table 3) [18,19]. Biofilm operons (*EpsA*, *TapA*, *SipW*, *TasA*), together with motility (*Fli*, *Mot*) and chemotaxis (*Che*) genes, enable effective root colonization and persistence (Figure 4). Secondary metabolite clusters, including *srf*, *itu*, *fen*, *pps*, *bac*, *alb*, and *dhb*, direct the synthesis of lipopeptides, antibiotics, and siderophores essential for pathogen suppression and nutrient acquisition [28,29,30]. Growth-promoting traits are encoded by *gcd* for phosphate mobilization, *ysnE* and *ipdC* for auxin production, and *AlsS* and *AlsD* for volatile organic compounds such as acetoin and 2,3-butanediol [56]. Additional pathways, such as *GabT* and *GabD*, contribute to cross-kingdom signaling via GABA metabolism [19]. Quorum sensing genes (*ComX*, and *Phr*) and two component systems (*DegS* and *DegU*; *PhoP* and *PhoR*) fine-tune these outputs in response to environmental and plant-derived cues [32]. Together, these loci form an integrated regulatory network that balances bacterial fitness with plant-beneficial outputs. This genetic versatility underpins the ecological success of *B. subtilis* and highlights its potential as a cornerstone of sustainable agriculture.

## 6. Summary and Future Perspectives

As a model plant-beneficial rhizobacterium, *B. subtilis* utilizes its genetic repertoire, including sporulation, robust biofilm formation, and the synthesis of antimicrobials and phytohormones, to promote plant growth and confer resistance against pathogens (Figure 1). This multifaceted function as a bio-fertilizer and biocontrol agent establishes it as a cornerstone for sustainable agriculture and forest management. To fully harness its potential and translate laboratory findings into practical applications, future research could shift towards more targeted and application-driven studies. Three examples are detailed as follows:

1. **Strain optimization for a specific application:** Moving beyond model strains, research should prioritize the isolation and characterization of novel *B. subtilis* isolate with enhanced, specialized traits. This includes screening for superior biocontrol activity against specific high-impact pathogens (e.g., *Fusarium wilt*, *R. solanacearum*) and identifying strains with high resilience to prevent biotic stresses like drought and soil salinity.
2. **Decoding the molecular dialog**: A deeper understanding of the specific molecular interaction is crucial. This requires employing omics studies such as transcriptomics and metabolomics to identify the key bacterial metabolites (e.g., specific lipopeptides, VOCs) and the corresponding plant genes they regulate. Elucidating this cross-kingdom signaling will allow for the rational design of more effective microbial consortia.
3. **Engineering effective microbial consortia**: Rather than relying on single-strain inoculants, the future lies in designing synthetic microbial communities. Research should unravel the synergistic interactions between *B. subtilis* and other native beneficial microbes (e.g., mycorrhiza fungi, nitrogen-fixers) to create stable, multifunctional consortia that provide compounded benefits for plant health and soil fertility.

## Data Availability

No new data were created or analyzed in this study. Data sharing is not applicable to this article.
